# Engineering the elongation factor Tu for efficient selenoprotein synthesis

**DOI:** 10.1093/nar/gku691

**Published:** 2014-07-26

**Authors:** Ken-ichi Haruna, Muhammad H. Alkazemi, Yuchen Liu, Dieter Söll, Markus Englert

**Affiliations:** 1 Department of Molecular Biophysics and Biochemistry, Yale University, New Haven, CT 06520-8114, USA; 2 Department of Chemistry, Yale University, New Haven, CT 06520-8114, USA

## Abstract

Selenocysteine (Sec) is naturally co-translationally incorporated into proteins by recoding the UGA opal codon with a specialized elongation factor (SelB in bacteria) and an RNA structural signal (SECIS element). We have recently developed a SECIS-free selenoprotein synthesis system that site-specifically—using the UAG amber codon—inserts Sec depending on the elongation factor Tu (EF-Tu). Here, we describe the engineering of EF-Tu for improved selenoprotein synthesis. A Sec-specific selection system was established by expression of human protein *O*^6^-alkylguanine-DNA alkyltransferase (hAGT), in which the active site cysteine codon has been replaced by the UAG amber codon. The formed hAGT selenoprotein repairs the DNA damage caused by the methylating agent N-methyl-N′-nitro-N-nitrosoguanidine, and thereby enables *Escherichia coli* to grow in the presence of this mutagen. An EF-Tu library was created in which codons specifying the amino acid binding pocket were randomized. Selection was carried out for enhanced Sec incorporation into hAGT; the resulting EF-Tu variants contained highly conserved amino acid changes within members of the library. The improved UTu-system with EF-Sel1 raises the efficiency of UAG-specific Sec incorporation to >90%, and also doubles the yield of selenoprotein production.

## INTRODUCTION

Ribosomal protein synthesis translates the information encoded on the mRNA into a nascent polypeptide according to the universal genetic code. The amino acids (aa) are first acylated onto their cognate tRNAs by aminoacyl-tRNA synthetases (aaRSs), thereby allowing elongation factors to bind and deliver aa-tRNAs to the ribosome (1). Of the 22 genetically encoded aa, Sec is the only one without a cognate aaRS. Bacteria employ a two-step mechanism for translational incorporation of Sec: (i) the tRNA^Sec^ is misacylated with serine by the endogenous seryl-tRNA synthetase (SerRS), and then (ii) the serine moiety of Ser-tRNA^Sec^ is converted to Sec by selenocysteine synthase (SelA) with selenophosphate (2).

Natural selenoprotein synthesis proceeds through co-translational insertion of Sec in response to a UGA opal codon. Two components are required for unambiguous Stop to Sec recoding. As Sec-tRNA^Sec^ is not recognized by the universal elongation factor Tu (EF-Tu), the specific elongation factor SelB discriminates against the misacylated Ser-tRNA^Sec^ and specifically binds Sec-tRNA^Sec^ (3,4). In addition, an RNA structural signal, the selenocysteine insertion sequence (SECIS) encoded immediately downstream of the UGA opal codon in bacteria, is also recognized by SelB. Only through ternary complex formation of Sec-tRNA^Sec^, SelB and SECIS-containing mRNA can a UGA opal codon be read unambiguously as Sec during ribosomal protein synthesis (5).

Recently, a synthetic amber suppressor tRNA^UTu^ (a tRNA for Sec [U] that is recognized by EF-Tu) was designed to be a substrate for SerRS, SelA and EF-Tu (6). *Escherichia coli* SelB deletion strains transformed with the components of the UTu system (the genes for tRNA^UTu^ and SelA) were capable of EF-Tu dependent selenoprotein synthesis where site-specific Sec insertion was directed by a UAG amber codon. Sec-tRNA^UTu^ is the product of a SelA-dependent conversion of Ser-tRNA^UTu^, an aminoacyl-tRNA that binds well to EF-Tu. Thus, should the tRNA-dependent transformation of Ser to Sec not be complete, both Sec and Ser will be incorporated by the UTu system. For instance, the tRNA^UTu^-mediated translation of *E. coli* glutaredoxin A mRNA (with UAG as codon 11) yielded 65% of selenoprotein, while the remainder was the Ser homolog (6).

EF-Tu weakly binds negatively charged aa, i.e. aspartate and glutamate (7,8). Since Sec is negatively charged under physiological conditions, a weak Sec-tRNA^UTu^–EF-Tu interaction can be expected. The opposite is true for SelB, which has a higher affinity for Sec-tRNA^Sec^ than for Ser-tRNA^Sec^ (4). The aim of this work is to create an EF-Tu variant that efficiently delivers Sec-tRNA^UTu^ to the ribosome. Using a Sec-specific selection system we generated such a variant that enhanced the amount of Sec incorporation and increased the selenoprotein product yield.

## MATERIALS AND METHODS

### Strains

The previously described *E. coli* BW25113 ΔselA, ΔselB, T7 RNA polymerase strain (*E. coli* MH3)—used for the UTu-mediated selenocysteine insertion system (6)—served as a parent strain to knock out the two endogenous alkyltransferases *ogt* and *ada* by the Datsenko and Wanner method (9). λ Red recombination with a kanamycin resistance cassette (from pKD4) harboring 50 nucleotide homologous sequences to the *ogt* genomic region at both ends allowed deletion of the *ogt* gene. The inserted kanamycin cassette contained two recognition sites for the FLP recombinase (from pCP20) which mediates the removal of the antibiotic cassette. This markerless BW25113 Δ*selA*, Δ*selB*, Δ*ogt*, T7RNAp strain was used for the removal of the *ada* gene with a kanamycin cassette containing a 200 nt homologous sequence to the *ada* genomic region. The final strain, *E. coli* BW25113 Δ*selA*, Δ*selB*, Δ*ogt*, Δ*ada*, T7RNAp, was designated *E. coli* KH1.

### Plasmids and libraries

The cloning of the plasmids used in this study is described in the Supplementary Data and below.

The coding sequence for the elongation factor Tu—representing the tufA gene—is a synthetic construct where the codons are manually changed in order to preserve codon usage but make the new coding sequence divergent from the tufA coding sequence (Supplementary Data). Hence, DNA oligonucleotides used for polymerase chain reaction do not target the genomic encoded tufA and tufB genes but only the synthetic tufA which is called orthogonal EF-Tu coding sequence (EF). EF was cloned into the NcoI and KpnI sites of pET-Duet1 (Novagen) and served as a template for overlap extension mutagenesis, in order to create the rationally designed EF-R1 (Y67, D216, R217, R274), EF-R2 (Y67, D216, R217, N274), EF-R3 (Y67, I98, D216, R217, R274) mutants and the EF library with the aa positions 67, 98, 216, 217 and 274 randomized as NNK-codons (N: GATC, K: GT). The assembled full-length coding sequences with the desired mutations were cloned into the NcoI and KpnI sites of pET-Duet1 (T7-promoter) by either T4 DNA ligation or the Gibson assembly kit (New England Biolabs). For the EF-library construction, a preparative T4 DNA ligation was performed using 10 μg each of EF coding sequence and pET-Duet1 (NcoI/KpnI hydrolyzed). 10^12^ *E. coli* XL-1 Blue cells were made electro-competent and used for the library transformation through electroporation. After 1 h of recovery in 2 l of 2 × terrific broth (TB) medium, ampicillin (100 μg/ml) was added for overnight growth. The cloning efficiency was tested by plating 2 μl of the recovered culture on lysogeny broth (LB) agar with ampicillin (10^8^ total colonies, 3 × 10^7^ codon diversity, 3 × 10^6^ amino acid diversity, 99% completeness). Additionally, some EF variants were excised from EF.PET with NcoI and KpnI and cloned into pBAD-myc-HisA (Invitrogen) (EF.BAD).

### Selenoprotein synthesis via the UTu-system

The UTu-system for selenoprotein overexpression used in this work included phosphoseryl-tRNA kinase (PSTK) (the eukaryotic kinase that converts Ser-tRNA^Sec^ into Sep-tRNA^Sec^) in order to decrease the amount of residual Ser-tRNA^UTu^ (6). The reporter proteins were the maltose binding protein fused with the Mxe gyrase A intein–chitin binding domain (MXB UAG_384_), the *Pyrococcus horikoshii* RNA ligase (RtcB UAG_98_), the *E. coli* thymidylate synthase (ThyA UAG_146_) and the *Bacillus subtilis* arsenate reductase (ArsC UAG_89_). *E. coli* MH3 cells were transformed with either MXB.RSF-UTU, RtcB.BAD-RSF-UTU, ThyA.RSF (with UTU.pGFib) or ArsC.RSF-UTU as well as SelA.PSTK.ACYC and optionally with EF.PET or EF.BAD variants. Typically, a 1 l main culture is supplemented with 10 μM selenite and the appropriate antibiotics (kanamycin 25 μg/ml, chloramphenicol 17 μg/ml and ampicillin 50 μg/ml (final concentrations)) and a 1/100 dilution of a pre-culture. After incubation at 37°C to an optical density of 0.8, temperature is shifted to 25°C and isopropylthiogalactoside (IPTG) is added to produce a final concentration of 0.1 mM. In the presence of arabinose-inducible constructs (RtcB.BAD-RSF-UTU and EF.BAD), arabinose is added to produce a final concentration of 0.005%. Cells were further incubated for 14–16 h (overnight), harvested by centrifugation and broken by sonification in Ni-NTA lysis buffer (20 mM HEPES, pH 7.7, 500 mM NaCl, 10 mM imidazole and optionally 10 mM 2-mercaptoethanol). Lysates were clarified by centrifugation (18 000 *g*, 60 min) and applied on Ni-NTA agarose (Qiagen) for a standard Ni-NTA purification with 200 mM imidazole elution. For the MXB.RSF construct, 2-mercaptoethanol was omitted from all buffers to preserve the uncleaved intact fusion of the maltose binding protein and the intein–chitin binding domain for subsequent analysis.

The ThyA-CBP-fusion protein was processed according to the IMACT-kit protocol (New England Biolabs). Cells were resuspended in lysis buffer (20 mM Hepes-NaOH, pH 8.5, 200 mM NaCl) and broken by sonification. The Chitin resin (New England biolabs) is loaded with the clarified lysate, washed with lysis buffer and then rapidly flushed with two column volumes of lysis buffer containing 50 mM dithiothreitol (DTT) for overnight (14–16 h) cleavage of the untagged ThyA protein from the resin-bound Mxe gyrase A intein–chitin binding domain. The eluted ThyA protein was further purified by the Mono Q HR5/5 column chromatography in Mono Q buffer (20 mM Tris-HCl, pH 8.5, 10 mM 2-mercaptoethanol) through a linear gradient over 20 ml of 0–500 mM NaCl. Fractions with a purity >95%—evaluated by Sodium dodecyl sulphate-polyacrylamide gel electrophoresis (SDS-PAGE)—were pooled and used for mass-spectrometric determination of the intact mass.

### Dithiothreitol-induced intein cleavage reaction of the MXB reporter

The MXB proteins from the Ni-NTA elution fraction were desalted on Sephadex G25 against 20 mM Na-HEPES, pH 8.5, 200 mM NaCl and 0.1 mM EDTA. The cleavage was initiated by addition of DTT (50 mM final). At the indicated times, 10 μl aliquots were quenched with SDS-loading dye (1 × conc.: 45 mM Tris-HCl, pH 6.8, 10% glycerol, 1% SDS, 0.01% bromophenol blue), stored on ice and directly loaded on the SDS polyacrylamide gel (without heat denaturation). All SDS gels were stained with Coomassie Blue.

### Western blot analysis of *PH* RtcB with anti-His(C-term)-HRP antibody

The SDS-PAGE separated proteins were electroblotted on a polyvinylidene difluoride (PVDF) membrane. An anti-His(C-term)-HRP antibody was applied according the manufacturer's instructions (Invitrogen). Chemiluminescence was initiated through the Western Lightning *Plus*-ECL reagent (Perkin Elmer) and visualized on a ChemiDoc MP station (BioRad).

### Selenocysteine-specific selection system

*E. coli* KH1 cells were transformed with AGT.BAD-RSF-UTU, SelA.PSTK.ACYC and EF.PET variants. Cells were grown in LB with 7.5 μM selenite, appropriate antibiotics, 0.1 mM IPTG and 0.1% arabinose at 30°C to an optical density of 0.6. Cells were pelleted by centrifugation (6000 *g*, 5 min), washed once with the same volume of 1 × M9 salts (Difco) and resuspended in the same volume of M9 salts with 10 μg/ml N-methyl-N′nitro-N-nitrosoguanidine (MNNG) for a 10 min incubation at room temperature. Cells were then pelleted and resuspended in the same volume of LB with 7.5 μM selenite, antibiotics, 0.1 mM IPTG and 0.1% arabinose for a 2-h recovery period at 30°C. The cycle of M9 salt wash, MNNG treatment and LB recovery was repeated for a total of three to five cycles before plating of 100 μl of a 10^−3^–10^−5^ dilution for rational-designed EF variants, or 100 μl (undiluted) for the library selection, on LB-agar with the above-mentioned supplements.

### Effect of EF-Tu mutants on phosphoserine-reporter expression

The fusion of the maltose binding protein with the superfolder green fluorescence protein encoding an UAG amber codon in position 2 was used as a reporter (MBP.GFP2TAG). The *E. coli* release factor 1 knockout strain EcAR7 (10) was transformed with the reporter MBP.GFP2TAG.*tac*RSF, SepRS9.pCAT-SepT and EF.BAD variants. Typically, 1 l of LB medium with antibiotics (25 μg/ml kanamycin, 17 μg/ml chloramphenicol, 50 μg/ml ampicillin) and 0.2 mM phosphoserine was inoculated at a ratio of 1/100 from a pre-culture. Cells were grown to an optical density of 0.6 at 34°C, then induced with IPTG and arabinose (0.2 mM and 0.005%, respectively) and incubated at 25°C for 14–16 h (overnight). The harvested cells were processed for standard Ni-NTA purifications as described in the selenoprotein reporter section. The ProQ Diamond stain of the SDS-polyacrylamide gel was performed according to the manufacturer's instructions (Invitrogen).

## RESULTS

### Establishing a selenocysteine-specific selection system

The development of orthogonal translation components is frequently based on read-through of an UAG amber codon in a reporter gene. However, in many cases, insertion of many different aa satisfy such a selection scheme (11). Ideally, one would like to develop an aa-specific selection strategy. Thus, we searched for a reporter gene that allows a Sec-specific selection. Of interest were enzymes containing active site Cys residues whose replacement with Sec (but not Ser) would lead to a functional enzyme, possibly with increased activity (12). A second requirement is that the original enzyme activity would protect against the selection drug. A literature search focused our attention on the human *O*^6^-alkylguanine-DNA alkyltransferase (hAGT) because (i) the hAGT protein contains an active site Cys which nucleophilically attacks the alkyl moiety of damaged DNA in a single turnover reaction and (ii) is the subject of an proven genetic selection for active variants from an hAGT library (13).

**Figure 1.**
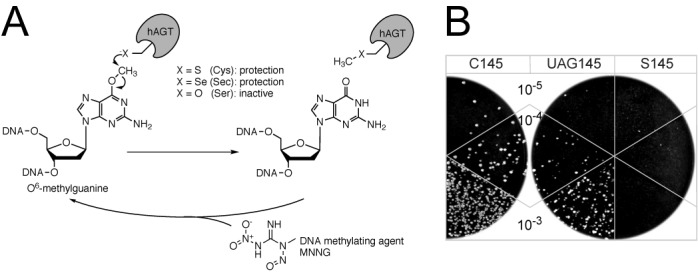
Overview of the Sec-specific genetic selection system. (**A**) *O*^6^-methylguanosine within DNA and human AGT with the active site residue at position 145 indicated as ‘X’. Whereas the hAGT C_145_ and U_145_ variants allow DNA repair, the hAGT S_145_ protein is inactive. The chemical structure of MNNG, which causes the conversion of guanosine to O^6^-methylguanosine in DNA, is shown. (**B**) *E. coli­* Δ*selA*, Δ*selB*, Δ*ada*, Δ*ogt*, T7RNAp cells were co-transformed with the UTu components and the hAGT C_145_, UAG_145_ and S_145_ variants. After three selection rounds with 10 μg/ml MNNG pulses and 2 h of recovery in-between, the indicated 10^−3^, 10^−4^ and 10^−5^ dilutions of cell suspensions were plated on LB agar to check for growth after 24 h.

To establish this system in *E. coli*, the two hAGT homologs *ada* and *ogt* were knocked out. The resulting cells were sensitive to the methylating agent MNNG. Any repair of MNNG-induced *O*^6^-methylguanine-DNA damage now requires the heterologous expression of hAGT variants. We tested the genetic selection system with the hAGT C_145_ (wild type) and S_145_, as well with the UTu-generated U_145_/S_145_ variants. Cells expressing hAGT, tRNA^UTu^ and SelA were subjected to three selection rounds with a 2-h recovery period at 30°C between the MNNG pulses. Next, sequential dilutions were plated on LB agar to obtain the fraction of surviving *E. coli* cells (Figure 1). While the hAGT C_145_ actively protected *E. coli*, expression of the hAGT S_145_ variant did not lead to cell survival when plated from a 10^−3^ dilution. A UAG codon (position 145) in hAGT mRNA is translated by the UTu system creating a mixture of hAGT U_145_ and S_145_ proteins—as confirmed by mass spectrometry of the purified mixture (Supplementary Figure S1). Visible growth comparable to that of wild-type hAGT indicates the active protection afforded by the hAGT U_145_ variant. Hence, any component of the UTu system that raises the amount of hAGT U_145_ protein is in the selected population.

### Engineering the amino acid binding pocket of EF-Tu

**Figure 2.**
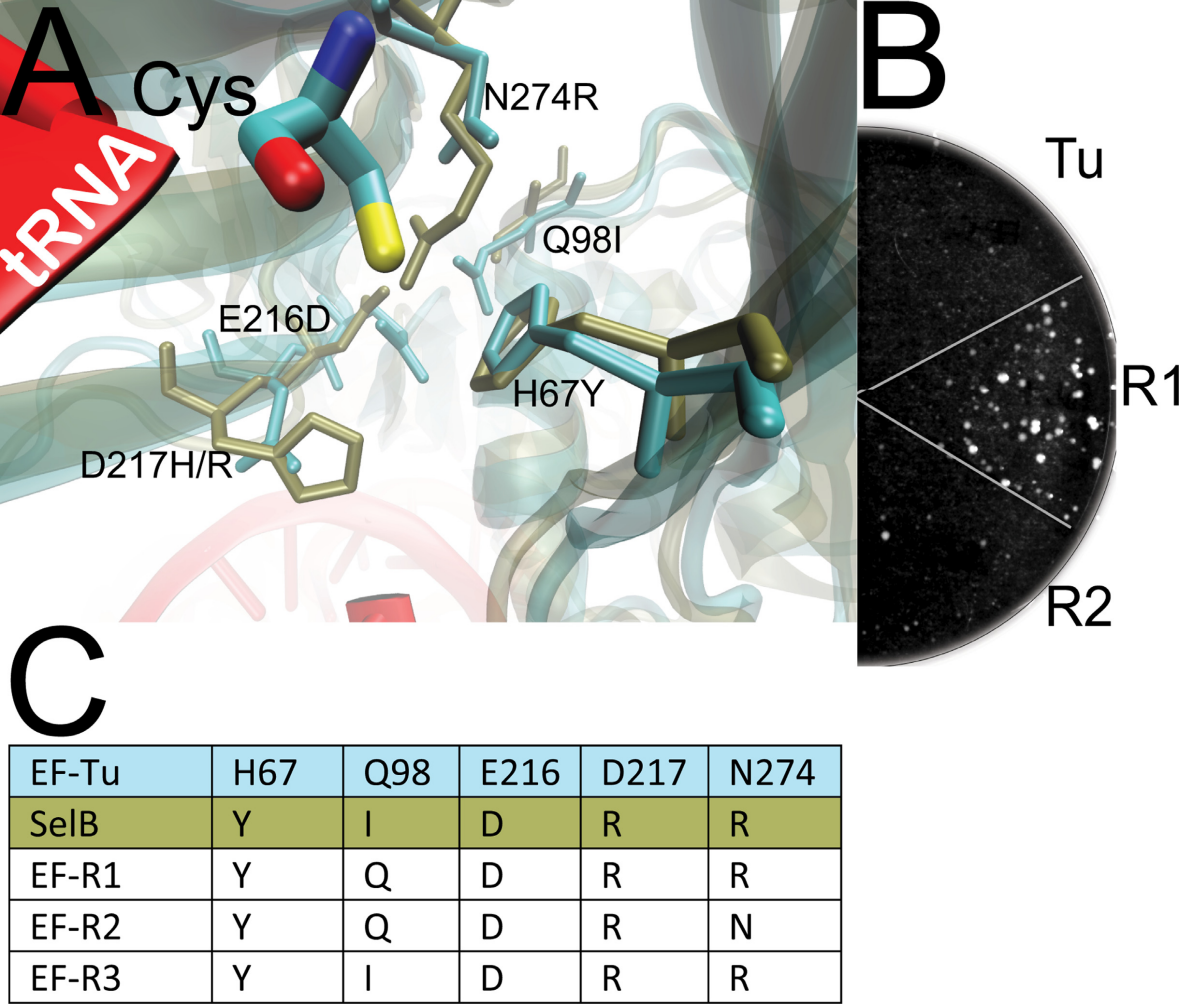
EF-Tu variant sequences and their effect on UTu-mediated selenoprotein formation. (**A**) The structure (10) of the ternary complex of EF-Tu (cyan) with GTP and Cys-tRNA^Cys^ (red and atomic coloring of Cys) is aligned to that (14) of SelB (brown). Residues forming the aa binding pocket are indicated using *E. coli* numbering. (**B**) hAGT UAG_145_ and the UTu components were supplemented with EF-Tu, EF-R1 and EF-R2 and subjected to three selection rounds before a 10^−5^ dilution was plated to indicate the surviving fraction. (**C**) Amino acid alignment of wild-type *E. coli* EF-Tu with the various EF-Tu variants and SelB.

The crystal structure (10) of the ternary complex of *Thermus aquaticus* EF-Tu, *E. coli* Cys-tRNA^Cys^ and GTP (PDB 1B23) was our guide for EF-Tu engineering (Figure 2). Residues near the aminoacyl moiety attached to the 3′ end of the tRNA make up the aa binding pocket of EF-Tu. Sec-tRNA^Sec^ is delivered by its own elongation factor, SelB, whose structure is known (PDB 4ACB) (14). A structural alignment of EF-Tu with SelB indicates a similar fold of both aa binding pockets, albeit with variations in residues forming a pronounced negatively charged surface on EF-Tu and, conversely, a positively charged surface on SelB (Figure 2). The alignment of several EF-Tu and SelB protein sequences highlights these differences: H67Y, E216D, D217R and N274R (using EF-Tu numbering).

Three variants were designed by transplanting residues from SelB into *E. coli* EF-Tu (EF-R1, EF-R2 and EF-R3; Figure 2). Cells co-expressing the EF-R variants, hAGT UAG_145_ and the other UTu components (tRNA^UTu^, SelA) were subjected to three rounds of MNNG treatment. Cells with EF-R1 allowed the highest hAGT U_145_ expression which protected most of the cells against MNNG; this was indicated by a >100-fold increase in colony number compared to cells having only wild-type EF-Tu. Hence, the delivery of Sec-tRNA^UTu^ by EF-Tu limits both the Sec/Ser ratio and the total yield of selenoprotein.

Inspired by the rationally designed EF-R proteins, a more detailed study on EF-Tu engineering was initiated. Transplantation of several residues could impact global folding and stability, simply by changing charge and space requirements due to the new residues. For example, the N274R mutation might be sterically hindered through Q98 in EF-Tu (Figure 2). An EF-Tu variant library was created by randomization of the aa in positions 67, 98, 216, 217 and 274 to NNK-codons.

### Selection of EF-Tu variant library results in variants with improved Sec incorporation

**Figure 3.**
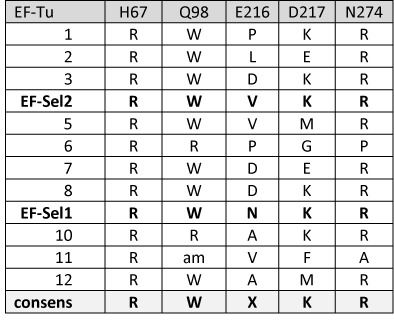
EF-Tu variants are listed which enabled *E. coli* to survive five Sec-selection rounds. A variant library was created with EF-Tu positions 67, 98, 216, 217 and 274 randomized as NNK-codons and selected for beneficial effects on UTu-mediated translation of hAGT/A_137_/UAG_145_.

The mutagen MNNG introduces many DNA replication errors, thereby facilitating the reversion of the hAGT/UAG_145_ to hAGT/C_145_ codons which would bypass the EF-Tu selection. As will be shown in the next paragraph, the hAGT/A137/U145 variant is fully active, while the hAGT/A137/C145 protein is inactive. Therefore, the hAGT/A137/UAG_145_ construct is used for the selection of the EF-Tu library with UTu components in *E. coli* (Δ*selA* Δ*selB* Δ*ada* Δ*ogt* T7RNAp). To fully select the diverse library, a total of five MNNG treatments with a higher drug dose (20 μg/ml) was necessary. From ≈10^9^ plated cells, 15–30 colonies grew, likely representing EF-Tu variants favoring the most efficient Sec-incorporation. The coding region of EF-Tu variants from 12 colonies were sequenced, resulting in one false-positive clone with an internal amber-stop codon at position 98. From the remaining 11 clones, all contained an R67, nine contained W98 and 10 R274, indicating these residues as being conserved. At position 217, there is a slight preference for Lys, whereas position 216 remained randomized (Figure 3). Position 98 was chosen to provide the compensatory space needed by R274; however, Trp is the bulkiest of the 20 standard aa. This contradiction can be explained with the assumption that W98 is flipped out of EF-Tu's aa binding pocket—like the Ile in the case of the Sec-specific elongation factor SelB (Figure 2). One clone with R98 and P274 was isolated. Here, the positive side chain of Arg reaches into the EF-Tu aa binding pocket from position 98 with space compensation provided by the small Pro at position 274.

Stable maintenance of the selected EF-Tu variants in pET-Duet1 was difficult in *E. coli* as their basal expression affected growth negatively. To overcome these toxic effects, two EF-Tu variants, EF-Sel1 and EF-Sel2, as well as wild-type EF-Tu and EF-Sep21 (15) were cloned under the arabinose promoter into the pBAD plasmid. Both variants, EF-Sel1 and EF-Sel2, share the conserved mutated residues R67, W98, K217 and R274 (Figure 3).

### Human AGT mechanism: Asn137 is responsible for Cys145 thiolate formation

**Figure 4.**
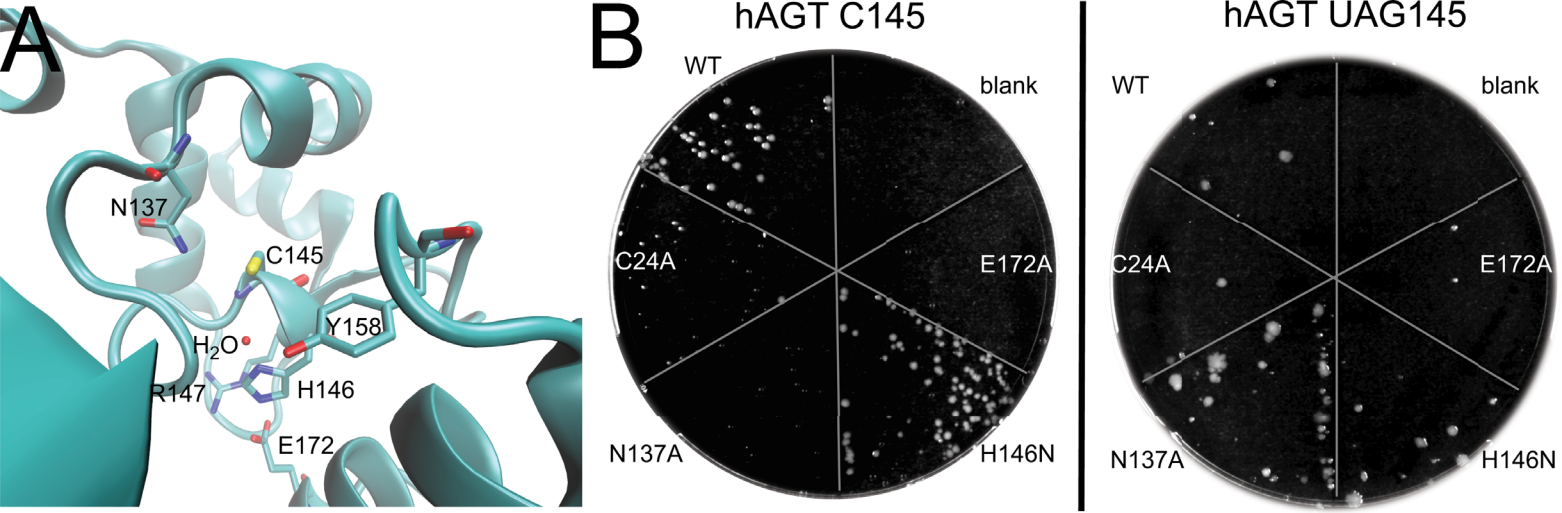
Active site of AGT and MNNG protection through hAGT variants with C_145_ and UAG_145_ mutations. (**A**) The crystal structure of *E. coli* alkyltransferase *ada* (pdb 1SFE) (17) reveals the residues and one ordered water molecule near the active site C_145_. The residue numbering corresponds to the positions in human AGT. (**B**) Four additional variations (C14A, N137A, H146N and E172A) were introduced as one set to the human AGT C_145_ and another set to UAG_145_ mutants. After three selection rounds with MNNG, a 10^−4^ dilution was plated to indicate growth.

The opportunity to make active Sec-containing hAGT variants led us to engage in a short mechanistic study. The accepted reaction mechanism of the hAGT protein requires the C_145_ thiol group to be assisted by nearby residues for deprotonation in order to form the active thiolate that then nucleophilically attacks the alkyl group of *O*^6^-alkylguanine in DNA (16). It was proposed (16) that the H146-E172 dyad deprotonates the C_145_ thiol group through a bridging water molecule (Figure 4). Additionally, the C_145_ thiol group is in direct contact with N137, but the function of this residue was unclear (16). We felt that a comparison of the properties of the hAGT Cys_145_ and Sec_145_ enzymes should clarify the hAGT mechanism. With a pKa of 5.5 Sec is present under physiological conditions as a selenolate, while Cys (pKa is 8.6) is a thiol. Guided by the structures (16,17), hAGT mutants in positions 24, 137, 146 and 172 were made. The individual variants C24A, N137A, H146N and E172A all contained the wild-type C_145_. Another set was created in which the variant had UAG_145_; this set was expected to show U insertion in the UTu system. Cells co-expressing the hAGT variants with the UTu-system in the presence of wild-type EF-Tu (without additional EF-Tu variants) were subjected to three rounds of MNNG selection before plating to reveal the surviving fractions (Figure 4). Surprisingly, the N146 mutant retained equal wild-type like activity for both hAGT C_145_ and U_145_ variants. In contrast, the A137 variant inactivated the hAGT C_145_ protein, but sustained the wild-type activity for the AGT U_145_ selenoprotein. Hence, the presence of N137 is important for hAGT/C145 activity, while the presence of H146 is not.

### Replacement of active site Cys with Sec in four reporter proteins

**Figure 5.**
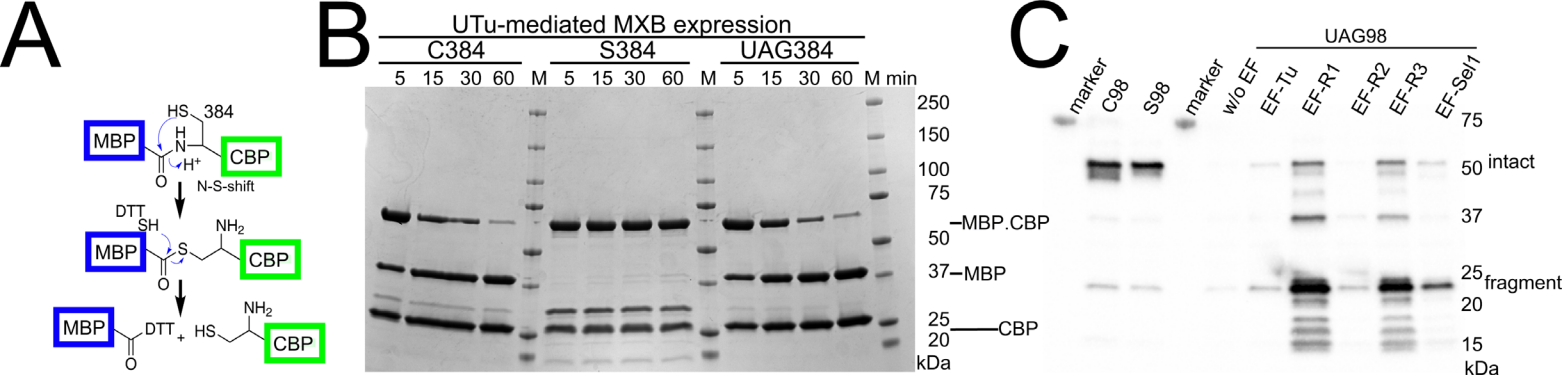
Reporter proteins MXB and RtcB characterization on SDS-PAGE. (**A**) The MXB protein (from NEB pMXB plasmid) is a fusion of an N-terminal MBP, linked through the *Mycobacterium xenopi gyrA* mini-intein to the CBP. In the presence of DTT, the intein mediates peptide cleavage into the two domains. (**B**) Three MXB variants of C_384_, S_384_ and UAG_384_ differ in the intein-cleavage active site. After Ni-NTA purification in the absence of reducing agents, DTT was added to a final concentration of 50 mM and aliquots were taken at the indicated time points. The cleavage patterns for the MXB variants are shown in the Coomassie Blue stained SDS-PAGE. (**C**) The *Pyrococcus horikoshii* RtcB C_98_, S_98_ and UAG_98_ variants are expressed with the UTu system in the absence or presence of the indicated EF-Tu variants for subsequent Ni-NTA purification. Aliquots of the Ni-NTA eluate are separated through SDS-PAGE and analyzed by western blot against the carboxyterminal His_6_-tag. The positions of intact RtcB C_98_/S_98_ proteins and the cleaved RtcB U_98_ fragment are indicated.

We tested our UTu system for Sec incorporation with four different proteins. The first two examples are of intein-mediated protein splicing. The first case was the control plasmid pMXB10 of the New England Biolabs IMPACT system. In this plasmid the coding region of the maltose-binding protein (MBP) is fused to the *Mxe* gyrase intein–chitin binding domain. Upon incubation of the full-length 70 kDa protein with DTT, the intein active site C_384_ cleaves the adjacent peptide bond to produce two fragments of MBP (42 kDa) and intein–chitin binding protein (CBP, 28 kDa). A similar cleavage is seen by the U_384_-containing protein; no significant cleavage activity has been seen for MXB S_384_ (Figure 5A). Judged by the cleavage pattern Cys and Sec are equally active.

The *Pyrococcus horikoshii* (*PH*) RNA ligase RtcB is the product of protein splicing (18,19) and Cys_98_ is the splice junction. However, if Sec replaces Cys_98_ (protein made in the UTu system), the more active Sec residue induces spontaneous protein cleavage of a nearby peptide bond regardless of the nature of the EF-Tu variant used for selenoprotein production (Figure 5C). The yield of RtcB UAG_98_ read-through is substantially raised in the presence of rationally designed EF-R1 and EF-R3—however, compromised with a 2-fold reduced growth rate of the production strain. The effect of the library selected EFSel1 is beneficial on the yield, albeit not to the same extent as EF-R1 or EF-R3.

The third case involved *E. coli* thymidylate synthetase (ThyA) where replacement of the active site Cys_146_ by Sec_146_ was genetically shown to produce an active enzyme (6). We synthesized ThyA U_146_ to subject it to intact mass spectrometry. The ThyA U_146_ (and the oxidized ThyA U_146_ protein with 2-mercaptoethanol bound) were represented with the highest intensity, and only a minor peak of the ThyA S_146_ mass could be detected (Supplementary Figure S2).

The last case was *Bacillus subtilis* arsenate reductase (ArsC/UAG_89_) to provide a second mass spectrometric example; it indicated the absence of ArsC S_89_ and the presence of ArsC U_89_ (Supplementary Figure S3).

In summary, four different reporters indicated at least 90% selenocysteine incorporation with the UTu system in response to the amber codon.

### EF-Sel and EF-Sep have different amino acid specificity

The work reported in this paper is our second case of EF-Tu engineering. We earlier showed that EF-Tu does not bind to phosphoseryl-tRNA, while selected EF-Tu variants (EF-Sep and EF-Sep21) are useful in mediating site-specific Sep insertion in *E. coli* (15,20). To see if EF-Sel can replace EF-Sep, both factors were individually added to the phosphoserine incorporation machinery for a reporter expression. Since EF-Sel2 did not lead to phosphoprotein production (Supplementary Figure S4), EF-Sel cannot substitute for EF-Sep.

## DISCUSSION

### Cellular fitness limits the range of engineering orthogonal translation systems.

The desire to expand the genetic code to produce proteins containing diverse aa (11,21) requires the development of orthogonal translation systems. Current experience suggests that optimal *in vivo* incorporation efficiency may require simultaneous engineering of tRNAs, aaRSs and elongation factors. Of the two known elongation factors, SelB and EF-Tu, the former one is orthogonal and recognizes a single aa-tRNA species, Sec-tRNA^Sec^ (4). The case is much different for EF-Tu which recognizes all other aa-tRNA species; this protein evolved for uniformly tight binding of the different aa-tRNAs engaging a complex mechanism (based on strong and weak binding properties) of the tRNA species and aa residues (22). Thus, EF-Tu engineering and genetic selection of variants is more complex than in the case of aaRSs of tRNAs, and may lead to protein variants with good properties for the desired aa residue, but overall toxic for *in vivo* protein synthesis because of damage to the interaction with other aa-tRNA species. Given the results described above, EF-R1 gave the best yield of the desired selenoprotein; however, the growth rate of the production strain was impaired. The genetic *in vivo* Sec-selection of the EF-Tu library took also cellular fitness into account; the resulting EF-Sel is a compromise between good selenoprotein yield and moderate toxicity. This may be similar to a recent report that reducing the selection pressure on a randomized aaRS library resulted in the isolation of different active aaRS variants (23).

### A vignette on human AGT mechanism

The *O*^6^-alkylguanine-DNA alkyltransferases are universally present in living organisms. Because of their role in maintenance of DNA integrity they are subject of many studies to develop resistance to cancer therapeutic alkylating agents (24). Regarding the enzyme mechanism it was deduced from the structure of the hAGT that N146-E172 dyad is responsible for deprotonating the active site Cys_145_ (16). However, our studies with several hAGT variants show N137 to be responsible for active site thiolate formation.

### Are selenoenzymes more active than their cysteine homologs?

Selenoproteins are essential in many organisms (25). Some selenoenzymes also have Cys homologs, which are generally considered to have lower catalytic efficiencies (12,26–27). As the results of Cys to Sec replacement are more complex than a simple pKa change (12,26–27) a general statement on the catalytic activity of selenoenzymes cannot be made. While it was shown that the Cys homolog of *E. coli* selenoenzyme formate dehydrogenase H is ∼5000-fold less active, the natural Cys homolog of *Drosophila* thioredoxin reductase shows similar activity to the human selenoenzyme (27,28). In this regard, our studies generated a hAGT N137A variant whose Cys_145_ form is inactive, while the Sec_145_ homolog is active.

## SUPPLEMENTARY DATA

Supplementary Data are available at NAR Online.

SUPPLEMENTARY DATA
