# Supplementary material for: Engineering the elongation factor Tu for efficient selenoprotein synthesis
Source: Nucleic Acids Res. 2014 Jul 26;42(15):9976–83. doi: 10.1093/nar/gku691 (PMC4150793; doi:10.1093/nar/gku691)
Supplement: SUPPLEMENTARY DATA [file supp_42_15_9976__index.html]

Engineering the elongation factor Tu for efficient selenoprotein synthesis — Engineering the elongation factor Tu for efficient selenoprotein synthesis — Engineering the elongation factor Tu for efficient selenoprotein synthesis — SUPPLEMENTARY DATA 

# Structural and kinetic insights into binding and incorporation of *L*-nucleotide analogs by a Y-family DNA polymerase

## SUPPLEMENTARY DATA

**Files in this Data Supplement:**

- SUPPLEMENTARY DATA
