# Supplementary material for: Engineering the elongation factor Tu for efficient selenoprotein synthesis
Source: Nucleic Acids Res. 2014 Jul 26;42(15):9976–83. doi: 10.1093/nar/gku691 (PMC4150793; doi:10.1093/nar/gku691)
Supplement: SUPPLEMENTARY DATA [file supp_gku691_nar-01305-r-2014-File007.docx]

Supplementary Data

**Engineering the elongation factor Tu for efficient selenoprotein synthesis**

Ken-ichi Haruna^1^, Muhammad H. Alkazemii^1^, Yuchen Liu^1^, Dieter Söll^1,2,*^ and Markus Englert^1,*^

^1^Department of Molecular Biophysics and Biochemistry and ^2^Department of Chemistry, Yale University, New Haven, CT 06520-8114, USA

*To whom correspondence should be addressed. Tel: +1 203 432 6206; Fax: +1 203 432 6202; Email: markus.englert@yale.edu

Correspondence may also be addressed to Dieter Söll. Tel: +1 203 432 6200; Fax: +1 203 432 6202; Email: [dieter.soll@yale.edu](mailto:dieter.soll@yale.edu)

**Plasmids**

The hAGT cDNA clone (MGC-5186) was ordered through ATCC and cloned into the NcoI and HindIII site of pBAD-myc-HisA (Invitrogen) adding a vector encoded His6-tag to the C-teminus of the hAGT protein. The pBR322 origin and ampicillin resistance marker of pBAD myc-HisA was replaced by the RSF-origin and kanamycin resistance marker from pRSF-Duet1 using the primer sets:

BAD_FSEF-CTCGTCGGCCGGCCGGAGCCTATGGAAAAACG,

BAD_NOTR-TAGAAAGCGGCCGCGGCCATCCGTCAGGATGG.

and RSF_NOTF-TAACCCGCGGCCGCCTCTAAACGGGTCTTG,

RSF_FSER-GAAATCGGCCGGCCCGGAATAGCTGTTCGTTGAC

Both PCR products were hydrolyzed with the restriction endonucleases FseI and NotI and subsequently joined with T4 DNA ligase. After ligation, the T4 DNA ligase was heat-inactivated and residual template DNA hydrolyzed by the restrictionendonuclease DpnI before the mixture was transformed into *E. coli* XL1-Blue. The resulting plasmid is called **AGT.BAD-RSF**.

For reporter expression experiments a codon-optimized cDNA of hAGT was cloned into the NcoI/KpnI hydrolyzed pRSF-UTU vector through Gibson assembly resulting in **rAGT-RSF-UTU**.

Using pGFIB-[tRNA^UTu^_am_] (1) as template, the lpp-promoter.tRNA^UTu^.rrnC-terminator region was amplified with GFINOTF-AAAAAAGCGGCCGCCCACCCTGGCGCCGCTTCTTTGAG and GFIKPNR-AAAAAAGGTACCGCTGGCGAAAGGGGGATGTGCTGC. AGT.BAD-RSF was used for inverse PCR amplification using BAD_NOTR and RSF_KPNF-AAAAAAGGTACCGGTCTTGAGGGGTTTTTTGCTGAAACCTC. Both PCR products were cloned together through their NotI and KpnI sites resulting in **AGT.BAD-RSF-UTU**. AGT.BAD-RSF-UTU was hydrolyzed with NcoI and HindIII and the vector backbone was ligated with the *Pyrococcus horikoshii* RtcB extein sequence (2) resulting in **RtcB.BAD-RSF-UTU**.

The coding region of the maltose binding protein fused in-frame to the coding region of the Mxe gyrase A intein-chitin binding domain was cloned from the pMXB10 plasmid (New England Biolabs) into the NcoI / PacI site of pRSF. A C-terminal His_6_ tag was inserted by inverse PCR, followed by blunt-end ligation, DpnI hydrolysis and transformation. This clone is called **MXB.RSF**. The lpp-promoter.tRNA^UTu^.rrnC-terminator region was amplified with UTU_FUSRSF_F- AAACCTCAGGCATTTGACGCCGCTTCTTTGAGCGA and UTU_FUSRSF_R- GTGACCGTGTGCTTCAAGCTTAAAAAAAATCCTTAGCTTTCGC, MXB.RSF was amplified by inverse PCR with RSF_UTU_F-GAAGCACACGGTCACACTG and RSF_UTU_R-TCAAATGCCTGAGGTTTCAG. Both PCR products were processed using the Gibson assembly kit (New England Biolabs) and the resulting plasmid is designated as **MXB.RSF-UTU**. *E. coli* thymidilate synthetase (ThyA) was amplified from genomic DNA with ECTY_NCO-AAAACCATGGAACAGTATTTAGAACTGATGC and ECTY_NOT-TTTTGCGGCCGCGATAGCCACCGGCGCTTTAATGC and cloned into the NcoI / NotI site of pTXB1 (New England Biolabs) to fuse the ThyA protein with the Mxe gyrase A intein/chitin binding domain for DTT induced tag removal. The scar sequence after the last residue of ThyA and before the protein-splicing active cysteine of the Mxe1 intein was removed by inverse PCR with ECTY_REV-GATAGCCACCGGCGCTTTAATGC and TXB_FOR-TGCATCACGGGAGATGCACTAG-3' for blunt-end ligation, DpnI hydrolysis and transformation. The coding sequence of ThyA-fusion protein was cloned into the NcoI / PacI sites of pRSF resulting in **ThyA.RSF**.

*Bacillus subtilis* arsenate reductase was amplified from genomic DNA with ArsC_NCO-GTTGCTCCATGGTTTCAACGAAAATACTTTGG and ArsCH6NOT-AAAAAAGCGGCCGCTTAATGATGATGATGATGATGTTTCCCTGTTTCAGCAAATTCCTTCAACC for subsequent cloning into the NcoI/NotI sites of pRSF-UTU resulting in **ArsC.RSF-UTU**.

The coding sequence for the superfolder green fluorescence protein (sfGFP, codon-optimized order through Genscript) was cloned into the NcoI / KpnI site of pRSF resulting in **sfGFP.RSF**. Subsequently, the second codon was changed to the TAG amber stop codon with the QuikChange procedure (**sfGFP2TAG.RSF**).

The coding sequence of the maltose binding protein (MBP) was amplified from the pMXB10 (New England Biolabs) with MBP_GFP_FW-GTTTAACTTTAATAAGGAGATATACATGGATATGAAAACTGAAGAAGG and MBP_GFPMRW-TAAACAGTTCTTCGCCCTT*cta*CATGTACTCGAGGAATTCGCG. The vector backbone of sfGFP2TAG.RSF was amplified with GFPMRSF_FW-ATG*tag*AAGGGCGAAGAAC and sGFPWRSF_RW-GTATATCTCCTTATTAAAGTTAAACAAAATTATTTCTAC and was used for Gibson assembly with the MBP-PCR product (**MBP.sfGFP2TAG.RSF**). The T7 promoter was replaced by the *tac*-promoter by inverse PCR on MBP.sfGFP2TAG.RSF with RSF_TAC_F-aattgtgagcggataacaatttcacacaggaaacagacCATGGATATGAAAACTGAAGAAGGTAAACTGG and RSF_TAC_R-ccacacattatacgggccggatgattaattgtcaacagctcATTTCCTAATGCAGGAGTCGC. After blunt-end ligation, the T4 DNA ligase was heat-inactivated at 65 °C for 15 min and residual template DNA hydrolyzed by DpnI before transformation into *E. coli* (**MBP.sfGFP2TAG.*tac*RSF**).

Side-directed mutagenesis leading to amino acid codon changes within the reporters AGT, RtcB, MXB and ThyA was performed according to the QuikChange procedure using Pfu Ultra II Fusion DNA polymerase in its 1x buffer (Agilent).

The plasmid with *E. coli* SelA in pACYC-Duet1 (through NcoI and NotI) served as template to insert the *E. coli* codon-optimized coding sequence for *Methanocaldococcus jannaschii* PSTK (through NdeI and PacI). The resulting construct is called **SelA.PSTK.ACYC**.

The engineered phosphoserine tRNA synthetase variant SepRS9 was amplified from pKD-SepRS9-EFSep21 (3) with SEPRS9NCOFW-GTTTTTTGGGCTAACAGGAGGAATTAACCATGGCAATGTTTAAAAGAGAAG and SEPRS9KPNRW-CAAGCTTCGAATTCCCATATGGTACCTTATTCAATTTTTACTTCGACATTTAAAAAG for Gibson assembly with the NcoI / KpnI hydrolyzed pBAD-myc-HisA vector resulting in **SepRS9.BAD**. The araC and SepRS9 region was amplified from SepRS.BAD with ARA_FWF_CAM-TTCGTGTCGCTCAAGGCGCACTCCCAAACGCCAGCAACGCGGC and ARA_RWF_CAM-TTTATCAGACCGCTTCTGCGTTCTGTTAGAAAAATAAACAAAAGAGTTTGTAGAAACGCAAAAAGGC and was used for Gibson assembly with the vector PCR product from the amplification of pCAT112TAG-SepT (4) with CAM_FW_ARA-CAGAACGCAGAAGCGGTCTG and CAM_RW_ARA-GGGAGTGCGCCTTGAGCG. The resulting plasmid is called **SepRS9.pCAT112TAG-SepT**. The amber stop codon within the chloramphenicol resistance gene was reverted to 112D via the QuikChange procedure resulting in **SepRS9.pCAT-SepT**.

**Protein characterization by mass spectrometry**

All MS analyses were performed by Keck MS & Proteomics Resource Laboratory at Yale University.

For the intact masses determination of the ThyA S146/U146 mixture, the Mono Q fraction was either used per-se, or additionally reduced with Tris(2-carboxyethyl)phosphine (TCEP). Briefly, the sample was desalted using C4 ZipTips, eluted in 60% acetonitrile / 0.1% formic acid and directly loaded on AB Sciex QSTAR Elite mass spectrometer. The intact masses of the *Bacillus subtilis* ArsC S89/U89 mixture were determined by FT-ICR MS on a Bruker FT MS APEX-Qe 9.4T instrument.

The hAGT S145/U145 mixture was reduced with DTT, alkylated by iodoacetamide, digested with trypsin and analyzed by LC-MS/MS on an Orbitrap Elite mass spectrometer. Mascot analysis was performed against the Swiss Prot database allowing Cys-by-Ser as well Cys-by-SecCarbamidomethyl replacement.

**Codon-optimized cDNA sequences:**

Recoded *E. coli* tufA cDNA: EF

ATGGCTAAGGAGAAATTCGAGCGCACTAAGCCACATGTCAACGTGGGCACCATTGGTCATGTAGATCACGGCAAGACTACCCTGACTGCGGCTATTACCACGGTTCTGGCCAAGACTTATGGTGGCGCGGCACGTGCGTTTGATCAGATTGACAATGCACCGGAGGAGAAGGCACGCGGCATTACAATTAACACCAGCCATGTAGAGTATGATACTCCGACTCGCCATTATGCGCATGTGGATTGTCCGGGCCATGCTGATTACGTAAAGAACATGATTACTGGCGCCGCACAGATGGATGGTGCTATCCTCGTCGTCGCAGCAACCGACGGTCCGATGCCACAGACCCGCGAACATATTCTGCTGGGCCGCCAGGTCGGTGTGCCTTATATTATCGTCTTTCTGAATAAGTGTGATATGGTAGACGATGAGGAACTGCTTGAGCTGGTAGAGATGGAGGTACGCGAGCTGCTGTCCCAGTATGATTTTCCGGGTGATGATACCCCGATTGTACGCGGCAGCGCCTTGAAGGCACTGGAGGGTGATGCGGAATGGGAGGCCAAGATTCTGGAGCTGGCGGGTTTTCTGGACAGCTATATCCCGGAGCCGGAACGCGCTATCGATAAACCGTTTCTGCTGCCGATTGAGGATGTGTTTAGCATTAGCGGCCGCGGCACTGTAGTCACTGGCCGCGTGGAGCGTGGCATCATTAAGGTAGGCGAGGAGGTCGAGATTGTGGGCATTAAGGAAACCCAGAAAAGCACTTGCACCGGTGTAGAGATGTTTCGTAAGCTGCTGGATGAGGGTCGCGCAGGCGAAAACGTGGGCGTACTCCTGCGTGGCATTAAGCGCGAGGAGATTGAGCGCGGCCAGGTTCTGGCCAAACCGGGTACAATTAAACCTCATACTAAATTTGAGAGCGAGGTTTATATCCTGTCTAAGGACGAGGGAGGTCGCCACACCCCATTTTTTAAGGGTTATCGCCCGCAATTTTATTTTCGCACCACCGATGTTACCGGCACTATCGAGCTGCCGGAGGGTGTTGAAATGGTTATGCCGGGTGATAACATTAAGATGGTAGTCACTCTGATTCATCCGATTGCGATGGATGATGGCCTGCGCTTTGCTATTCGCGAGGGTGGTCGCACTGTAGGTGCTGGTGTGGTTGCGAAGGTGCTCTCATAA

Codon optimized *Methanocaldococcus jannaschii* PSTK cDNA:

ATGGGCGATATTATGCTGATTATTCTGACCGGTCTGCCTGGTGTTGGTAAAAGCACCTTTAGCAAAAATCTGGCCAAAATCCTGAGCAAAAACAACATTGATGTTATTGTGCTGGGCAGCGATCTGATTCGTGAAAGTTTTCCGGTTTGGAAAGAGAAATACGAAGAGTTTATCAAAAAAAGCACGTACCGCCTGATTGATAGCGCACTGAAAAACTATTGGGTGATTGTGGATGATACCAACTACTATAATAGCATGCGTCGTGACCTGATCAACATTGCCAAAAAATACAACAAAAACTACGCCATCATCTATCTGAAAGCAAGCCTGGATGTGCTGATTCGTCGTAATATTGAACGCGGTGAAAAAATTCCGAACGAAGTGATCAAAAAAATGTACGAAAAATTCGATGAACCTGGGAAAAAATACAAATGGGATGAACCGTTTCTGATCATCGATACCACCAAAGATATCGACTTTAACGAAATCGCCAAAAAACTGATCGAAAAAAGCAAAGAAATCCCGAAATTCTACGTGCTGGAAGAAAACAAAAACAAAAATAACAACATCAGCGATAAAATCGATAAAGAAACCCGCAAAATCGTGAGCGAGTACATCAAAAGTAAAAAACTGGATAAAGACAAAATCAAAGAAGTGGTCGAACTGCGCAAAGAATTTCTGAAAAAAATCAAAAAAATGGAAGAGGTGGATGCCGATCGTGTGCTGAAAGAATTTAAAGATCTGCTGAACAGCTATTGA

Codon optimized superfolder green fluorescence protein cDNA:

ATGAGCAAGGGCGAAGAACTGTTTACGGGCGTGGTGCCGATTCTGGTGGAACTGGATGGTGATGTCAATGGTCACAAATTCAGCGTGCGCGGCGAAGGTGAAGGCGATGCAACCAATGGTAAACTGACGCTGAAGTTTATTTGCACCACGGGTAAACTGCCGGTTCCGTGGCCGACCCTGGTCACCACGCTGACGTATGGTGTTCAGTGTTTCAGTCGTTACCCGGATCACATGAAACGCCACGACTTTTTCAAGTCCGCGATGCCGGAAGGTTATGTCCAAGAACGTACCATCTCATTTAAAGATGACGGCACCTACAAAACGCGCGCCGAAGTGAAATTCGAAGGTGATACGCTGGTTAACCGTATTGAACTGAAAGGCATCGATTTTAAGGAAGACGGTAATATTCTGGGCCATAAACTGGAATATAACTTCAATTCGCACAACGTGTACATCACCGCAGATAAGCAGAAGAACGGTATCAAGGCTAACTTCAAGATCCGCCATAATGTGGAAGATGGCAGCGTTCAACTGGCCGACCACTATCAGCAAAACACCCCGATTGGTGATGGCCCGGTCCTGCTGCCGGACAATCATTACCTGAGCACGCAGTCTGTGCTGAGTAAAGATCCGAACGAAAAGCGTGACCACATGGTCCTGCTGGAATTCGTGACCGCGGCCGGCATCACGCACGGTATGGACGAACTGTATAAAGGCTCACATCATCATCATCATCATTGA

Codon optimized human AGT cDNA:

ATGGATAAAGATTGCGAGATGAAACGTACCACCCTGGATAGTCCGCTGGGTAAACTGGAACTGAGCGGTTGTGAACAGGGTCTGCATGAAATTAAACTGCTGGGCAAAGGCACCAGCGCAGCAGATGCAGTTGAAGTTCCGGCACCGGCAGCAGTTCTGGGTGGTCCGGAACCGCTGATGCAGTGTACCGCATGGCTGAATGCATATTTTCATCAGCCGGAAGCCATTGAAGAATTTCCGGTTCCTGCCCTGCATCATCCGGTTTTTCAGCAAGAAAGCTTTACCCGTCAGGTTCTGTGGAAACTGCTGAAAGTTGTTAAATTTGGCGAGGTGATTAGCTATCAGCAGCTGGCAGCACTGGCAGGTAATCCGAAAGCAGCACGTGCAGTTGGTGGTGCAATGCGTGGTAATCCGGTTCCGATTCTGATTCCGTGTCATCGTGTTGTTTGTAGCAGCGGTGCCGTTGGTAATTATTCAGGTGGTCTGGCAGTTAAAGAATGGCTGCTGGCACATGAAGGTCATCGTCTGGGCAAACCGGGTCTGGGTGGCAGCAGTGGACTGGCTGGTGCGTGGCTGAAAGGTGCCGGTGCAACCAGCGGTAGCCCTCCGGCAGGTCGTAATCATCATCACCATCATCATTAA

**
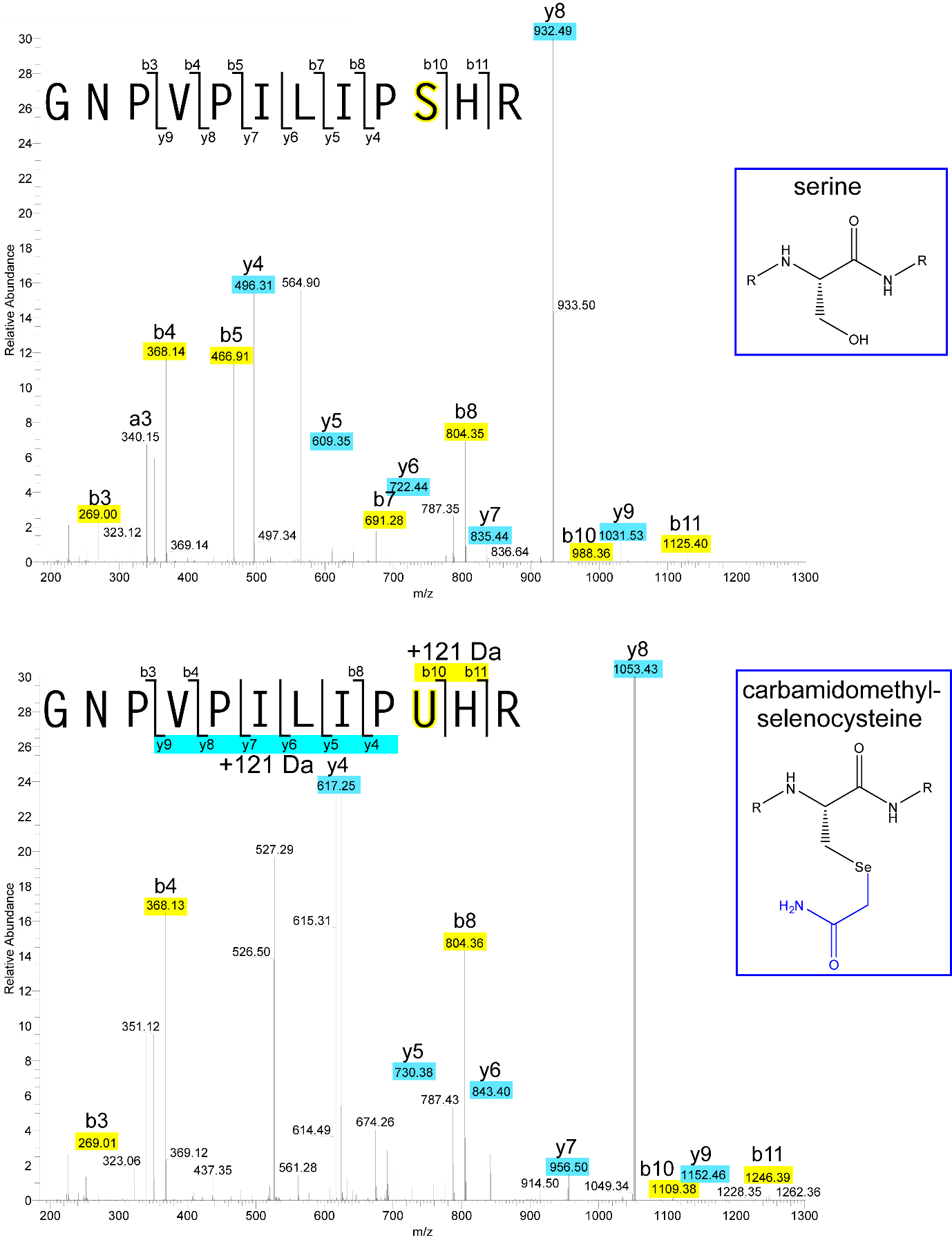
**

B

A

**Figure S1.** LC-MS/MS analysis of the tryptic hydrolysate of the hAGT S145/U145 mixture. (**A**) The tryptic peptide resulting from the UAG_145_ read-through by serine is shown. Secondary fragmentation ions from the b- and y-series are indicated. (**B**) Incorporation of selenocysteine (alkylated by iodoacetamide prior analysis) in response to the UAG_145_ amber codon shift the masses of the b10, b11, as well as y4, y5, y6, y7, y8 and y9 ions by 121 Da compared to the serine-containing peptide.

B

**
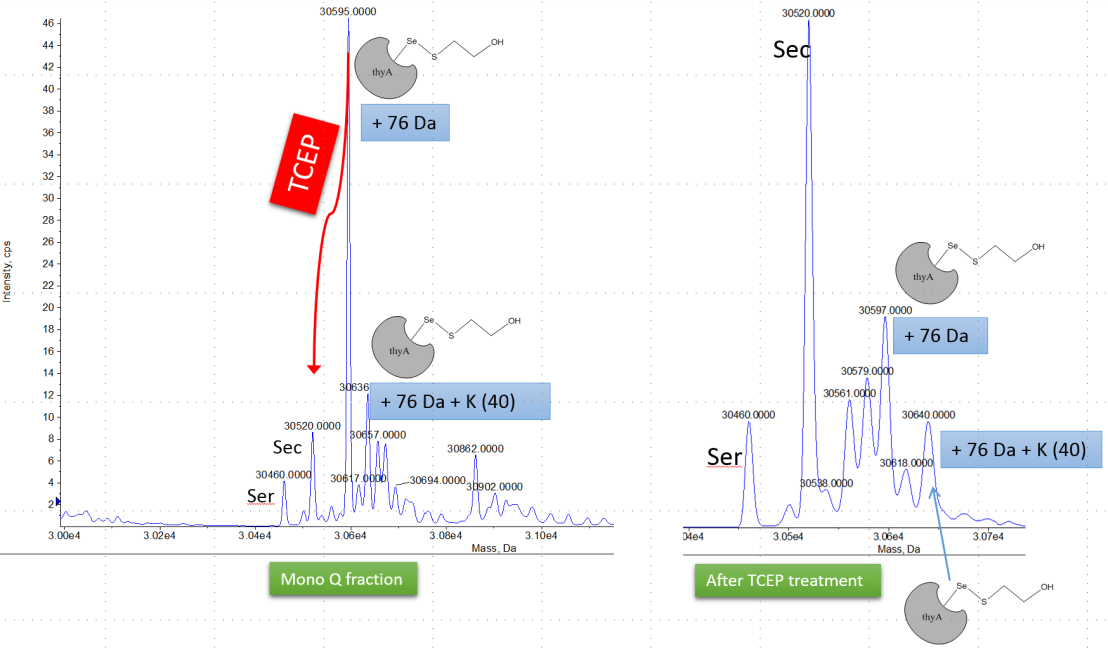
**

**Figure S2.** Intact masses of the *E. coli* ThyA S_146_/U_146_ mixture was determined by ESI/MS. (**A**) The UTu-generated ThyA S_146_/U_146_ mixture was purified over Chitin resin and Mono Q chromatography and subsequently used for intact mass determination by ESI-MS. Peaks are labeled with the determined mass in Da. Calculated molecular weight for ThyA S_146_ is 30464 Da, for ThyA U_146_ 30527 Da. Likely, most of the ThyA U_146_ protein got oxidized and has bound 2-mercaptoethanol resulting the putative ThyA U_146_-2ME peak of 30603 Da. (**B**) The ThyA protein mixture was incubated with the strong reducing agent Tris(2-carboxyethyl)phosphine (TCEP) and the subjected to ESI/MS. While the putative ThyA U_146_-2ME peak of 30603 Da got reduced, the ThyA U_146_ peak increased; this is an indication for the reversible ThyA U_146_ selenol oxidation with 2-mercaptoethanol.

Ser

Sec

+ 76 Da

+ 76 Da + K (40)

After TCEP treatment

Mono Q fraction


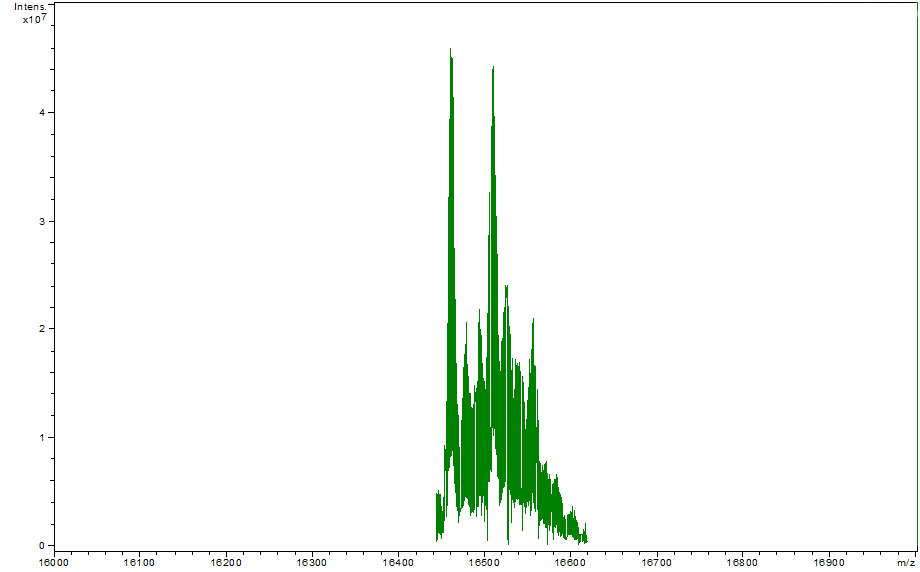


Sec + K 16505

Sec 16465

Ser 16402

**Figure S3**. Intact masses of the *Bacillus subtilis* ArsC S89/U89 mixture were determined by FT-ICR MS. The ArsC/UAG_89_ construct was expressed with the UTU-system and EF-Sel1. The resulting proteins were purified over Ni-NTA and analyzed by FT-ICR MS. No corresponding peak could be observed for the ArsC/S89 variant (16402 Da). The ArsC/U89 variant is represented per-se (16465 Da) and with a potassium ion bound (16505 Da).

16465 + 40

Sec + K

16402

Ser

16465

Sec


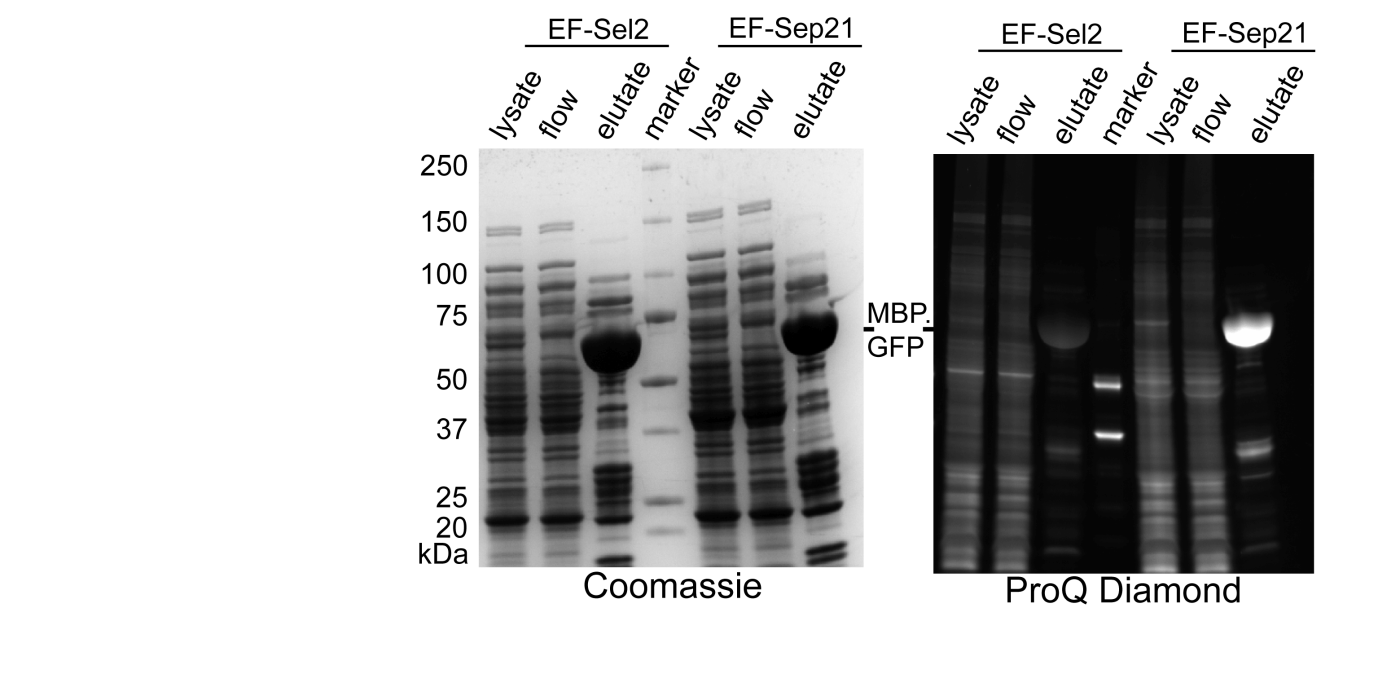

**Figure S4.** Effect of the EF-Tu variants EF-Sel2 and EF-Sep21 on co-translationally phosphoserine (Sep) insertion. The Sep incorporation machinery of SepRS9 and tRNA^Sep^ was co-expressed with either the selenocysteine-selected EF-Sel2 or the phosphoserine-selected EF-Sep21 in the release factor 1 knock out strain EcAR7 (5). The standard Ni-NTA purification was documented by separating aliquots of the clarified lysate, flow through after Ni-NTA and imidazole elution through SDS-PAGE. Proteins were visualized by Coomassie Blue (left panel), phosphoproteins by the ProQ Diamond (right panel) staining. The position of the MBP.GFP reporter of 70 kDa is indicated. For the right panel the PeppermintStick Phosphoprotein marker (Invitrogen) was used showing the intensity of 1 µg of the double phosphorylated ovalbumin and β-casein. *E. coli* contains about 80 native phosphoproteins which are also visible in the lysate and flow through lanes of the ProQ Diamond stain. Without an efficient orthogonal phosphoserine incorporation system (through the absence of EF-Sep21), read-through of the UAG amber codon likely occurs through near-cognate suppression due to the knockout of release factor 1 in the EcAR7 strain.

**LITERATURE**

1. Aldag, C., Bröcker, M.J., Hohn, M.J., Prat, L., Hammond, G., Plummer, A. and Söll, D. (2013) Rewiring translation for elongation factor Tu-dependent selenocysteine incorporation. *Angewandte Chemie*, **52**, 1441-1445.

2. Englert, M., Xia, S., Okada, C., Nakamura, A., Tanavde, V., Yao, M., Eom, S.H., Konigsberg, W.H., Söll, D. and Wang, J. (2012) Structural and mechanistic insights into guanylylation of RNA-splicing ligase RtcB joining RNA between 3'-terminal phosphate and 5'-OH. *Proceedings of the National Academy of Sciences of the United States of America*, **109**, 15235-15240.

3. Lee, S., Oh, S., Yang, A., Kim, J., Söll, D., Lee, D. and Park, H.S. (2013) A facile strategy for selective incorporation of phosphoserine into histones. *Angewandte Chemie*, **52**, 5771-5775.

4. Park, H.S., Hohn, M.J., Umehara, T., Guo, L.T., Osborne, E.M., Benner, J., Noren, C.J., Rinehart, J. and Söll, D. (2011) Expanding the genetic code of Escherichia coli with phosphoserine. *Science*, **333**, 1151-1154.

5. Heinemann, I.U., Rovner, A.J., Aerni, H.R., Rogulina, S., Cheng, L., Olds, W., Fischer, J.T., Söll, D., Isaacs, F.J. and Rinehart, J. (2012) Enhanced phosphoserine insertion during Escherichia coli protein synthesis via partial UAG codon reassignment and release factor 1 deletion. *FEBS letters*, **586**, 3716-3722.
